# Glycolytic Reprogramming Mediated by the OTUD4-FXR1-HIF1A Axis Drives Gastric Cancer Progression

**DOI:** 10.7150/ijbs.138384

**Published:** 2026-09-03

**Authors:** Fulin Wang, Yu Shao, Xiaomei Zhuang, Xiumin Liu, Yu Hu, Chen Dai, Jia Chen, Yong Kuang, Gangqiang Wang, Zhenzhen Zhao, Nan Cai, Liang Gu, Guoying Zhou, Hongwu Chu, Huimin Zhao, Cuncan Deng, Changhua Zhang

**Affiliations:** 1 Gastrointestinal Surgery, Digestive Diseases Center, The Seventh Affiliated Hospital, Sun Yat-sen University, Shenzhen, China.; 2 Guangdong Provincial Key Laboratory of Digestive Cancer Research, The Seventh Affiliated Hospital, Sun Yat-sen University, Shenzhen, China.; 3 Scientific Research Center, The Seventh Affiliated Hospital of Sun Yat-sen University, China.; 4 Department of Gastrointestinal Surgery, The Eighth Affiliated Hospital, Sun Yat-sen University, Shenzhen, China.

## Abstract

Ubiquitination and deubiquitination play critical roles in gastric cancer progression, yet systematic investigations of ubiquitin-regulating proteins in gastric cancer (GC) remain limited. To identify key regulators, we performed Ubiquitin-Focused CRISPR-Cas9 growth-based screening in GC cells and identified the deubiquitinase OTUD4 as a candidate gene promoting tumor growth. Functionally, OTUD4 was found to act as an oncogene by promoting the proliferation and metastasis of GC cell. Mechanistically, OTUD4 interacts with FXR1 through its N-terminal region (1-245 aa), and mutation of the catalytic cysteine residue (C45A) markedly impairs this interaction. OTUD4-mediated deubiquitination removes K48-linked polyubiquitin chains from FXR1, thereby protecting FXR1 from proteasomal degradation and increasing its stability. The resulting accumulation of FXR1 enhances HIF1A mRNA stability, leading to increased HIF1A expression. Consequently, HIF1A activates glycolytic programs and promotes the malignant progression of gastric cancer. Clinically, OTUD4 expression was significantly higher in tumor tissues, and higher OTUD4 levels were linked to decreased overall survival. Importantly, OTUD4 and FXR1 expression levels show a positive correlation in human GC samples. Collectively, these findings uncover an OTUD4-FXR1-HIF1A signaling axis that promotes glycolysis, which in turn functionally drives gastric cancer progression, and thereby provides a potential prognostic biomarker and therapeutic target.

## Introduction

Gastric cancer (GC) is still one of the top causes of cancer-related deaths around the world 1. Although both incidence and mortality have declined in recent years and treatment options now include surgery, chemotherapy, targeted agents, and immunotherapy, GC continues to pose a substantial clinical challenge 2-4. The limited efficacy of available treatments in advanced disease is an important reason for this burden. A clearer understanding of the molecular events driving GC progression, together with identification of critical regulatory molecules, is therefore essential for developing more effective targeted treatments and improving patient outcomes.

Ubiquitination is a reversible post-translational process that controls protein stability and signaling by covalently attaching ubiquitin to target proteins 5. Dysregulation of this system has been linked to diverse diseases, including malignancies, neurodegenerative disorders, and inflammatory conditions, highlighting ubiquitin signaling as an important regulatory network and therapeutic opportunity 6-8. A body of evidence suggests that ubiquitination and deubiquitination are important in the progression of gastric cancer. Increasing evidence indicates that ubiquitination-related processes contribute to GC progression. For instance, TRIM17 facilitates BAX ubiquitination and proteasomal degradation, thereby limiting BAX-dependent apoptosis and supporting gastric cancer growth and survival 9. TRIM24 promotes GC proliferation and metastasis through NRBP1 ubiquitination 10. RNF167 and STAMBPL1, an E3 ligase and a deubiquitinase, respectively, regulate Sestrin2 polyubiquitination and connect leucine availability with mTORC1 inhibition, suggesting potential therapeutic relevance in cancer 11. While awareness of DUBs' involvement in tumorigenesis and progression is increasing, a systematic study of other DUBs' participation in gastric cancer has not been completed.

Here, we performed a screening system based on CRISPR/Cas9 (clustered regularly interspaced short palindromic repeats/CRISPR-associated protein 9) to identify functional DUBs in promoting gastric cancer progression. We identified OTU deubiquitinase 4 (OTUD4) as a key regulator of gastric cancer progression. Mechanistically, we found that OTUD4 promotes activation of the hypoxia inducible factor 1, alpha subunit (HIF-1α) signaling pathway by regulating FXR1 expression, thereby enhancing tumor cell glycolysis and ultimately facilitating tumor growth and metastasis. This research extends our grasp of the role ubiquitination-related proteins play in gastric cancer progression and supplies mechanistic insights for the creation of OTUD4-targeted therapeutic strategies.

## Materials and Methods

### Patients and tissue samples

Matched gastric cancer and adjacent non-tumorous tissues were collected from patients undergoing surgical resection at the first and seventh affiliated hospitals of Sun Yat-Sen University. Eight pairs of fresh specimens were immediately stored at -80°C for subsequent RNA or protein extraction. For IHC and mIHC analyses, 208 paraffin-embedded GC specimens and 20 paired adjacent normal samples were prepared. All samples were pathologically diagnosed as gastric adenocarcinoma. Clinical and pathological information was obtained from the corresponding medical records (Table S1). None of the enrolled patients had received chemotherapy or radiotherapy before surgery.

### Cell lines and cell culture

Human gastric cancer cell lines (HGC-27, SNU-719, MKN-45) and human embryonic kidney cells (293T) were obtained from the Chinese Academy of Sciences (CAS) Shanghai Cell Bank and cultured in RPMI-1640 or DMEM medium, enriched with 10% fetal bovine serum (FBS) and 1% penicillin/streptomycin, at 37°C in a humidified incubator with 5% CO₂. Mycoplasma contamination was routinely checked.

### Animal experiment

In all mouse experiments conducted, the maximum permitted tumor volume of 1600 mm³ was not exceeded. A pathogen-free environment was used to maintain the mice, with conditions set at 20 ± 2 °C for temperature, 55 ± 10% for humidity, and a 12-hour light/dark cycle. 4-week-old BALB/c nude mice were acclimated to the animal facility for one week prior to experimentation. Subcutaneous xenograft and peritoneal metastasis mouse models were established to evaluate the *in vivo* effects of OTUD4 and FXR1 on tumor proliferation and peritoneal dissemination. For subcutaneous tumor formation, a suspension containing 1 × 10⁶ control or genetically modified MKN45 cells in 100 μL of PBS was injected subcutaneously into the flank of each mouse. For the peritoneal metastasis model, 5 × 10^5^ control or genetically modified MKN45 cells suspended in 100 μL of PBS were injected intraperitoneally. Mice were sacrificed 4 weeks after injection, and tumors or peritoneal metastatic nodules were collected for further analysis.

### Plasmid and lentiviral transfection

Cells were plated in suitable culture dishes and allowed to grow until they reached 60-70% confluence for plasmid transfection. According to the manufacturer's guidelines, plasmids were introduced using Lipofectamine 3000 (Thermo Fisher, Waltham, MA, USA). After 6 to 8 hours, the medium was changed to fresh complete medium, and the cells were cultured for another 40 to 42 hours.

For lentiviral transduction, cells were infected with lentiviruses carrying control or target genes in the presence of 8 μg/mL polybrene (Beyotime, Shanghai, China). After 24 h, the viral supernatant was replaced with fresh medium, and stable cell lines were selected using the appropriate antibiotic according to the resistance marker encoded by the lentiviral vector. shRNA sequences are provided in Table S2.

### Ubiquitin-focused CRISPR-Cas9 growth-based screening

#### Ubiquitination-related CRISPR-Cas9 library screening

A focused CRISPR-Cas9 loss-of-function screen was performed using the Human Ubiquitination-Related Proteins CRISPR Knockout Library, an all-in-one lentiviral CRISPR library. This library contains 11,108 sgRNAs targeting 660 ubiquitination-related genes, including major components of the ubiquitin system such as E1 ubiquitin-activating enzymes, E2 ubiquitin-conjugating enzymes, E3 ubiquitin ligases, and deubiquitinating enzymes 12. Lentiviral particles were produced in HEK293T cells by co-transfecting the sgRNA library plasmids with psPAX2 and pMD2.G packaging plasmids. The lentiviral library was used to infect gastric cancer cells at a low MOI (≤ 0.3) to guarantee that each cell received only one sgRNA.

#### Puromycin selection and long-term growth screening

Forty-eight hours after infection, cells were selected with puromycin for 5-7 days to eliminate non-transduced cells. Following selection, a portion of the cells was harvested as the baseline sample (D0). The remaining cells were continuously cultured under standard growth conditions for 14 days, while maintaining sufficient cell numbers to preserve a library coverage of at least 500-fold. At day 14 (D14), cells were collected for genomic DNA extraction. Changes in sgRNA abundance between D14 and D0 were used to identify ubiquitination-related genes involved in the regulation of long-term cell growth and survival.

#### sgRNA sequencing and data analysis

Genomic DNA was isolated with the TIANamp Genomic DNA Kit (TIANGEN, Beijing, China). sgRNA regions were amplified in two PCR steps and sequenced on an Illumina platform. After alignment of sequencing reads to the reference library, differential sgRNA abundance between D0 and D14 was quantified with MAGeCK. Genes showing significant sgRNA enrichment or depletion at a false discovery rate below 0.05 were defined as candidate regulators.

### Quantitative reverse-transcription PCR (qRT-PCR)

The FastPure Cell/Tissue Total RNA Isolation Kit V2 (Vazyme, Nanjing, China) was used to extract total RNA. Subsequently, equal quantities of RNA were transformed into cDNA with the help of the PrimeScript RT-PCR Kit (Accurate Biotechnology, Hunan, China). Quantitative real-time PCR was then performed with SYBR Green I (Vazyme) as the fluorescent dye. The 2^-ΔCt method was employed to calculate relative gene expression levels, which were normalized to *ACTB*. Table S3 contains the primer sequences used in this study.

### Western blotting

Total cellular proteins were extracted using RIPA lysis buffer (Beyotime) containing protease and phosphatase inhibitors, and protein concentrations were measured using a BCA protein assay. Total protein in equal amounts was resolved by 10% SDS-PAGE and subsequently transferred onto PVDF membranes (Millipore, Burlington, MA, USA). The membranes were treated with the specified primary antibodies (as detailed in Table S4), then incubated with suitable secondary antibodies. The detection of protein signals was carried out with a ChemiDoc imaging system (Bio-Rad, Hercules, CA, USA), and β-actin or LMNB1 was employed as a loading control.

### Cell proliferation

Cell proliferation was measured with the Cell Counting Kit-8 (CCK-8, Biosharp, Hefei, China). Cells were placed in 96-well plates at a suitable density and left to adhere overnight. At the indicated time points, a volume of 10 μL of CCK-8 solution was added to each well, followed by a 2-hour incubation at 37°C. The absorbance at 450 nm was measured using a microplate reader.

### Plate colony formation

Transfected cells were grown in the previously described media for 10-14 days after being seeded into 6-well plates at a density of 1000 cells per well for the colony-formation experiment. The resultant colonies were dyed with 0.1% crystal violet after being fixed with 4% paraformaldehyde (PFA). The colonies were counted and photographed after staining.

### Cell cycle assays

Cell cycle assays were performed according to the manufacturer's instructions (Beyotime). Cells were subjected to fixation with ethanol overnight followed by staining with propidium iodide in conjunction with RNase A. A CytoFlex LX Flow Cytometer (Beckman Coulter, Brea, CA, USA) was used to conduct the analysis. For the assessment of cell cycle, FlowJo software (V10, FlowJo, Treestar, OR, USA) was employed.

### Wound-healing assay

Once the monolayer reached 80-90% confluence in six-well plates, a sterile pipette tip was used to generate a scratch. Serum-free medium was maintained during the assay to minimize the contribution of cell proliferation. Images were acquired at 0 and 48 h, and the corresponding changes in wound area were quantified with ImageJ (V1.8.0).

### Transwell migration assay

Transwell chambers with 8 μm pores (Corning, NY, USA) were used to assess cell migration. Cells were suspended in a medium without serum and placed in the upper chamber, while the lower chamber received medium with 10% FBS to serve as a chemoattractant. Following a 24-hour incubation, cells that did not migrate were wiped off the upper membrane with a cotton swab. The cells that moved to the lower side were fixed with 4% PFA, stained with 0.1% crystal violet, and visualized using a Leica DMi8 microscope (Leica, Wetzlar, Germany). Cells that migrated were counted in at least three random areas per well.

### Transwell invasion assay

The ability of cells to invade was assessed using Transwell chambers that were pre-coated with Matrigel (Corning). To summarize, Matrigel was diluted in a medium without serum and spread evenly on the upper side of the Transwell membrane following the manufacturer's instructions, then incubated at 37°C for 4 hours to allow for Matrigel polymerization before adding cells. The gastric cancer cells were deprived of serum and then placed in a medium without serum. An equal number of cells were seeded into the upper chambers. The bottom chambers were filled with a complete medium that included 10% fetal bovine serum. After incubation for 48 h, cells were fixed with methanol and stained with 0.2% crystal violet. Invaded cells were imaged, while the number of invaded cells was quantified from randomly selected fields.

### RNA sequencing

Total RNA was extracted from OTUD4 control or knockdown SNU-719 cells and used for RNA sequencing. Library preparation and sequencing were performed by LC-Bio Technology Co., Ltd. (Hangzhou, China).

### Untargeted metabolomics study

Untargeted metabolomics analysis was performed by LC-Bio Technology Co., Ltd. Briefly, the cells were gathered, quickly frozen in liquid nitrogen for an hour, and then kept at -80 °C. Metabolite extraction, LC-MS data acquisition, and subsequent bioinformatics analysis were carried out by the service provider according to their standard protocols.

### Glycolytic activity assays

Lactate concentrations in culture supernatants were measured with a Lactate Assay Kit (Beyotime) following the manufacturer's guidelines and adjusted based on cell count.

Glucose uptake was evaluated using the glucose uptake kit (Beyotime). Glucose uptake was quantified based on the mean fluorescence intensity (MFI).

ECAR was measured using a Seahorse XF Extracellular Flux Analyzer (Agilent Technologies, Santa Clara, CA, USA) following the manufacturer's protocol. Cells were placed in Seahorse XF plates to undergo a glycolysis rate assessment. The number of cells was used to normalize the ECAR values.

### Immunoprecipitation (IP) and mass spectrometry (MS)

Cells with proteins tagged with FLAG or Myc were broken down using ice-cold IP lysis buffer. Equal amounts of protein lysates were incubated with anti-FLAG or anti-Myc magnetic beads (MedChemExpress, NJ, USA) overnight at 4°C with gentle rotation. Proteins that were bound were eluted using SDS sample buffer by boiling, preparing them for immunoblotting and mass spectrometry. LC-Bio Technology Co., Ltd. conducted the mass spectrometry analysis.

### Immunofluorescences (IF)

Immunofluorescence staining was performed as previously described 13.

### RNA immunoprecipitation (RIP)

The RNA Immunoprecipitation (RIP) experiment was carried out utilizing anti-FXR1 and anti-IgG antibodies. This process was performed using the Magna RIP RNA-binding protein immunoprecipitation kit (Millipore), adhering strictly to the guidelines provided by the manufacturer. The enrichment values obtained were adjusted to account for the background levels of RIP identified through the use of the IgG isotype control.

### RNA pulldown

The RNA Pulldown Kit (Huijun Biotech, Shanghai, China) was used to conduct RNA pulldown assays. Briefly, biotin-tagged RNA probes were produced by Sangon Biotech (Table S5, Shanghai, China). The probes were incubated with cell lysates in the provided binding buffer for 2 h. The kit's streptavidin magnetic beads were used to capture RNA-protein complexes, and subsequent washing steps removed nonspecific bindings. Bound proteins were eluted and analyzed by SDS-PAGE and immunoblotting.

### Immunohistochemistry (IHC) and multiplex immunohistochemistry (mIHC)

Paraffin sections (4 μm) were deparaffinized in xylene and passed through graded ethanol for rehydration. Antigen retrieval was carried out in EDTA buffer (pH 6.0, Maxim, Fuzhou, China) by microwave treatment for 15 min, followed by blockade of endogenous peroxidase with 3% hydrogen peroxide for 10 min. For IHC, sections were incubated with primary antibodies (Table S4) at 4°C overnight and then with HRP-conjugated secondary antibodies for 1 h at room temperature. DAB was used for signal development, and hematoxylin served as the nuclear counterstain. For mIHC, repeated cycles of primary antibody staining and TSA amplification were performed according to the manufacturer's instructions (Aifang Biological, Changsha, China). Fluorescence images were acquired with a KF-FL-120 digital pathology slide scanner (KFBIO, Ningbo, China).

### Bioinformatic analysis

#### Bulk RNA sequencing analysis

RNA sequencing data from both public databases and our experiments were analyzed. Differential expression analysis between tumor and normal tissues was performed using DESeq2 in R. ClusterProfiler facilitated the execution of pathway enrichment analyses, including GO and KEGG. Results were visualized with heatmaps, volcano plots, and other appropriate plots.

#### Survival analysis

In the Kaplan-Meier survival analysis, patients were grouped into high and low OTUD4 expression categories, determined by the median OTUD4 expression level within the cohort. The Kaplan-Meier method and the log-rank test were used to compare overall survival (OS) between the two groups. The prognostic value of OTUD4 expression was assessed using both univariate and multivariate Cox proportional hazards regression analyses. The clinicopathological variables included sex, age, Borrmann classification, tumor grade, T stage, and N stage, together with OTUD4 expression status. In the Cox regression analyses, low OTUD4 expression, female sex, age <60 years, Borrmann type I, poor tumor grade, T 1&2 stage, and N 0&1 stage were used as the reference categories, respectively. Hazard ratios (HRs) and 95% confidence intervals (CIs) were calculated.

### Statistics analysis

All data were analyzed using SPSS 20.0 software (IBM SPSS Statistics, Armonk, NY, USA) or R software (version 4.5.0). Comparisons between two groups were performed using unpaired or paired two-tailed Student's t-tests, as appropriate. Comparisons among three or more groups were conducted using one-way ANOVA or two-way ANOVA followed by Tukey's multiple comparisons test. Categorical data were analyzed using the chi-square (χ²) test. Correlations between variables were assessed using Pearson or Spearman correlation coefficients, depending on data distribution. The number of independent biological replicates (N) is indicated in each figure legend. Data are presented as mean ± SD. *P < 0.05, **P < 0.01, ***P < 0.001, ns = not significant.

## Results

### CRISPR-based functional genomic screening identifies OTUD4 as a promoter of gastric cancer progression

To systematically identify new therapeutic targets for gastric cancer (GC), We first used a lentiviral Ubiquitin-Focused CRISPR-Cas9 library targeting human ubiquitination-related proteins to search for genes that regulate GC growth (Fig. 1A) 12. Before downstream analysis, the quality of the screening library and sequencing data was assessed. The sgRNA library showed high coverage with robust representation, and biological replicates exhibited strong concordance, supporting the reliability and reproducibility of the screening system (Fig. S1A-D). In addition, known regulators of GC growth, including *TRIM28*, *KDM2A*, and *USP7*, were negatively selected in the screen, further validating the efficiency and specificity of our screening approach (Fig. 1B, Table S6) 14-16. Among genes associated with GC-cell survival and proliferation, OTU deubiquitinase 4 (OTUD4) was notable because its cancer-related function remains controversial and it showed the strongest sgRNA depletion in our screen. We then created stable OTUD4 knockdown cells in two gastric cell lines using shRNA. The depletion efficiency of OTUD4 was verified at both mRNA and protein levels (Fig. 1C, D). CCK8, plate colony formation, and cell cycle assays were carried out to evaluate the effect of OTUD4 on GC cell proliferation. As expected, the cell viability and the number of cell colonies were reduced in OTUD4 knockdown group (Fig. 1E-G). Moreover, we observed that deficiency of OTUD4 induced cell cycle arrest mainly in the G2/M phase, which is consistent with previous report in glioblastoma (Fig. 1H, I) 17. In addition, we performed RNA sequencing analyses with OTUD4 knockdown and control SNU-719 cells (Table S7). Gene set enrichment analysis (GSEA) indicated that the positive regulation of epithelial cell proliferation showed lower enrichment scores in OTUD4 knockdown SNU-719 cells (Fig. S2A). *In vivo*, subcutaneous implantation of OTUD4-knockdown MKN45 cells into BALB/c nude mice produced smaller tumors with lower weights than control cells (Fig. 1J-L). IHC further showed a reduced proportion of Ki67-positive cells in OTUD4-deficient xenografts (Fig. S2B). Surprisingly, knockdown of OTUD4 did not affect tumor cell apoptosis, as neither the apoptosis rate nor the levels of apoptosis-related proteins showed significant changes between groups (Fig. S2C-S2E). These findings suggest that OTUD4 contributes to gastric cancer progression *in vitro* and *in vivo*.

### OTUD4 promotes cell migration, invasion, and peritoneal dissemination in GC

Our RNA seq data also showed that the migration was downregulated in OTUD4 knockdown SNU-719 cells (Fig. S3A). Consistent with this finding, OTUD4 knockdown lowered the mesenchymal markers N-cadherin and Vimentin while increasing E-cadherin (Fig. S3B). We therefore assessed cell motility and invasion directly. Wound closure was slower after OTUD4 depletion (Fig. S3C), and both Transwell migration and invasion assays showed impaired motile and invasive behavior (Fig. S3D, E). Because peritoneal spread represents a common metastatic pattern in GC 18, we evaluated the potential for peritoneal metastasis following OTUD4 knockdown in GC cells, using the model we previously reported 19. The number and volume of macro metastatic nodules were significantly reduced in the OTUD4 knockdown group compared to control group (Fig. S3F, G). Overall, these findings support a role for OTUD4 in promoting metastatic phenotypes of gastric cancer.

### OTUD4 drives gastric cancer progression by promoting HIF1A-dependent glycolytic reprogramming

We next examined how OTUD4 drives GC progression. RNA-seq revealed broad transcriptional changes after OTUD4 depletion (Fig. 2A, B). GSEA revealed that glycolysis/gluconeogenesis pathway ranked among the top enriched pathways and glycolytic activity was significantly downregulated in OTUD4-deficient GC cell lines (Fig. S4; Fig. 2C). The non-targeted metabolomics displayed that lactate, which is the end product of glycolysis, was decreased in OTUD4 knockdown SNU-719 cells (Fig. 2D, E). Moreover, the extracellular lactate level was lower in OTUD4 knockdown GC cells than control group (Fig. 2F). Additionally, the flow cytometry displayed that OTUD4 knockdown GC cells had a decreased glucose uptake level (Fig. 2G). Seahorse analysis revealed a marked reduction in the extracellular acidification rate (ECAR) following OTUD4 knockdown (Fig. 2H). We proceeded to analyze the expression of enzymes that play a crucial role in glycolysis. Consistent with these functional changes, the expression level of proteins such as glucose transporter 1 (GLUT1), lactate dehydrogenase A (LDHA), and Alpha-enolase (ENO1), required for glycolysis, were dramatically decreased in OTUD4 deficiency GC cells (Fig. 2I). Thus, OTUD4 appears to positively regulate glycolytic metabolism in GC.

Because glycolytic activity is closely linked to malignant progression, we asked whether OTUD4 promotes GC partly through metabolic activation 20. RNA sequencing revealed that the HIF1 signaling pathway, a key regulator of glycolysis, was significantly downregulated in the OTUD4 knockdown group (Fig. 2J). Since HIF1A serves as the master regulator of the HIF1 signaling cascade, we next investigated whether OTUD4 modulates HIF1A expression and activity. Results of quantitative real time PCR (qRT-PCR) and western blotting (WB) confirmed that HIF-1α expression was reduced in OTUD4 knockdown group (Fig. 2K, L). Therefore, we overexpressed HIF1A in both control and OTUD4-knockdown gastric cancer cells and subsequently assessed their proliferative and metastatic capacities. We next restored HIF1A expression in control and OTUD4-knockdown GC cells. HIF1A overexpression increased GLUT1, ENO1, and LDHA abundance in both backgrounds (Fig. S5A) and elevated lactate production (Fig. S5B). It also enhanced proliferative and metastatic phenotypes in control as well as OTUD4-deficient cells (Fig. S5C, D). Taken together, these findings indicate that OTUD4 contributes to gastric cancer progression through HIF1A-dependent glycolytic reprogramming.

### FXR1 acts as a key mediator linking OTUD4 to glycolytic reprogramming

We then sought to determine how OTUD4 controls glycolysis. Because OTUD4 is a DUB and its depletion altered HIF1A expression, we first tested whether HIF1A itself might be a direct OTUD4 substrate. However, coimmunoprecipitation (Co-IP) failed to detect an OTUD4-HIF-1α interaction (Fig. S6A). We thus performed mass spectrometry (MS) analysis to identify potential OTUD4 substrates and identified FXR1, an RNA-binding protein, as one of the significantly enriched candidates (Table S8, S9). Notably, FXR1 has been reported to potentially interact with HIF-1α based on omics-based analyses 21. Co-IP assays showed that FXR1 could physically bind to OTUD4 in HGC27 and SNU-719 cells (Fig. 3A). We also performed immunofluorescence (IF) experiments to detect the co-expression pattern of FXR1 and OTUD4 and found that FXR1 was largely co-expressed with OTUD4 (Fig. 3B). Moreover, FXR1 protein expression was significantly reduced following OTUD4 knockdown (Fig. 3C). A CHX chase experiment further demonstrated faster FXR1 turnover after OTUD4 knockdown, indicating that OTUD4 contributes to FXR1 stability (Fig. 3D, E). Treatment with the proteasome inhibitor MG132 restored FXR1 abundance (Fig. 3F), implicating proteasomal degradation. Consistently, polyubiquitinated FXR1 accumulated after OTUD4 depletion (Fig. 3G). OTUD4 overexpression selectively reduced K48-linked FXR1 ubiquitination, with no clear effect on K63-linked or the other tested atypical linkages (K6, K11, K27, K29, and K33) (Fig. 3H). Domain mapping analysis indicated that the N-terminal OTU motif of OTUD4 (aa 1-245) mediates their interaction. Mutation of OTUD4 at its catalytic site (C45A) reduced its ability to bind FXR1, suggesting that the interaction between OTUD4 and FXR1 partially depends on the integrity of OTUD4's catalytic domain (Fig. S6B). In summary, these results indicated that OTUD4 maintains FXR1 levels by regulating K48-linked polyubiquitination.

We next investigated whether OTUD4 enhances glycolytic activity by regulating FXR1. We found that lactate production, glucose uptake and ECAR were partially recovered when FXR1 was overexpressed in OTUD4-deficient GC cells (Fig. 3I; Fig. S7A, B). In addition, WB analysis showed that GLUT1, LDHA and ENO1 levels were increased upon FXR1 overexpression in OTUD4-deficient GC cells (Fig. 3J). These findings identify FXR1 as a functional downstream effector of OTUD4-mediated glycolytic regulation.

### OTUD4 promotes GC progression in a FXR1-dependent manner

We next examined whether FXR1 accounts for the effects of OTUD4 on GC proliferation and metastasis. FXR1 was overexpressed in control and OTUD4-deficient cells. Relative to the empty-vector condition, increased FXR1 enhanced cell proliferation and colony formation (Fig. 4A-C). Wound-healing, Transwell migration, and invasion assays similarly showed greater migratory and invasive activity after FXR1 overexpression, irrespective of OTUD4 status (Fig. 4D-I). Thus, restoring FXR1 partially compensated for the impaired motility and invasion caused by OTUD4 depletion *in vitro*. *In vivo*, FXR1 overexpression also increased tumor growth and metastatic dissemination in OTUD4-deficient models (Fig. 5A-E). Consistent with these observations, mIHC showed a higher proportion of Ki67-positive cells after FXR1 restoration (Fig. 5F, G), further supporting FXR1 as a functional mediator of OTUD4-driven GC progression.

### FXR1 promotes glycolysis reprogramming by stabilizing *HIF1A* mRNA

To establish whether FXR1 mediates OTUD4-dependent regulation of HIF1A, we generated FXR1-knockdown GC cells and confirmed depletion at the mRNA and protein levels (Fig. 6A, B). HIF-1α expression was subsequently reduced in both HGC-27 and SNU-719 cells after FXR1 knockdown (Fig. 6C, D). Given the RNA-binding properties of FXR1, we proposed that it might regulate HIF1A through direct association with its transcript. RNA immunoprecipitation (RIP) assays showed enrichment of HIF1A mRNA in FXR1 immunoprecipitates relative to IgG controls (Fig. 6E-G). We then generated wild-type and mutant biotinylated RNA probes based on RBPsuite predictions (Fig. 6H; Tables S5, S10) 22. RNA pulldown experiments demonstrated binding of FXR1 to the wild-type probe but not the mutant probe (Fig. 6I). To assess transcript stability, actinomycin D was used to inhibit new RNA synthesis. The results showed that FXR1 depletion accelerated *HIF1A* mRNA decay compared with controls (Fig. 6J). Together, the data indicate that FXR1 associates with HIF1A mRNA and helps maintain its stability.

We next asked whether HIF1A represents the principal functional effector downstream of FXR1. HIF1A was ectopically expressed in FXR1-deficient GC cells, followed by assessment of glycolysis and malignant phenotypes. Restoring HIF1A increased GLUT1, ENO1, and LDHA expression in FXR1-knockdown cells (Fig. S8A) and elevated lactate production, indicating recovery of glycolytic activity (Fig. S8B). Importantly, HIF1A re-expression also counteracted the inhibitory effects of FXR1 depletion on proliferation and metastatic behavior (Fig. S8C, D). These results support the conclusion that FXR1 promotes GC progression largely by sustaining HIF1A-dependent glycolytic reprogramming.

### OTUD4 is upregulated in GC and predicts poor clinical outcomes

We next assessed the clinical relevance of OTUD4 and FXR1 using TCGA data. Both genes showed significantly higher expression in GC than in normal tissues (Fig. 7A-D), and OTUD4 levels were further increased in stage III-IV tumors relative to stage I-II disease (Fig. 7E). Analysis of eight freshly collected GC specimens confirmed higher OTUD4 expression in tumors than in paired adjacent normal tissues at both the RNA and protein levels (Fig. 7F, G). IHC of 20 paired clinical samples provided additional evidence of OTUD4 upregulation in GC tissue (Fig. 7H-J). In a cohort of 208 patients, higher OTUD4 expression was associated with shorter overall survival in Kaplan-Meier analysis (Fig. 7K). Univariate Cox analysis also identified OTUD4 as a prognostic factor (Fig. S9A), and the association remained significant after adjustment for age, sex, Borrmann classification, tumor grade, T stage, and N stage in multivariate analysis (Fig. S9B). We next performed multiplex immunohistochemistry (mIHC) analysis and found that OTUD4 and FXR1 co-localize in gastric cancer tissues, and their expression levels are positively correlated (Fig. 7L, M). In addition, higher OTUD4 expression was also accompanied by a greater proportion of Ki67-positive cells (Fig. 7N).

## Discussion

In this study, we identified OTUD4 as a regulator of gastric cancer progression through a CRISPR-based screening approach. OTUD4 is an OTU-family deubiquitinase previously implicated in DNA damage responses, innate immunity, and apoptosis 23-28. However, its role in metabolic regulation and GC progression had remained largely unexplored. Our data show that OTUD4 promotes GC growth and dissemination by stabilizing the RNA-binding protein FXR1, which enhances HIF1A signaling and glycolytic activity. The increase in key glycolytic enzymes offers energy and biosynthetic intermediates that facilitate the proliferation, migration, and invasion of tumor cells. Importantly, OTUD4 deficiency impairs these processes, while FXR1 overexpression partially rescues them, revealing a previously unrecognized OTUD4-FXR1-HIF1A axis linking deubiquitinase-mediated regulation to metabolic reprogramming in gastric cancer.

OTUD4 has been reported to exert context-dependent functions in different tumor types, suggesting that its role in cancer is highly dependent on cellular and molecular backgrounds. In liver cancer, OTUD4 cooperates with IRTKS to enhance SETDB1-mediated H3K9 trimethylation, which suppresses E-cadherin expression and facilitates metastasis 29. In glioblastoma, OTUD4 drives tumor progression through CDK1 deubiquitination and MAPK activation 17. It also drives an immunosuppressive microenvironment in spinal metastases of triple-negative breast cancer through the OTUD4-ZMYND8-DDX3X axis, supporting tumor growth and dissemination 30. In addition, OTUD4 can stabilize GPX4 and suppress autophagic degradation, thereby reducing ferroptosis and favoring tumor-cell survival 24. Conversely, tumor-suppressive functions have been described in colorectal cancer, where OTUD4 stabilizes p53 through deubiquitination 31. In clear cell renal cell carcinoma, OTUD4 stabilizes RBM47, which promotes ATF3 transcription and restricts malignant phenotypes 32. Beyond protein stabilization, OTUD4 also regulates cell death. It enhances the sensitivity of nasopharyngeal carcinoma to radiation through GSDME-mediated pyroptosis and influences DNA repair to make non-small cell lung cancer more receptive to radiation 26, 28. Collectively, these observations emphasize the context-dependent nature of OTUD4 biology. Our findings add GC to this spectrum and identify OTUD4 as a tumor-promoting factor that acts through FXR1 stabilization and HIF-1α-dependent glycolytic reprogramming.

Our study found that the HIF-1α pathway was suppressed in OTUD4-deficient gastric cancer cells. HIF-1α drives glycolysis and multiple malignant hallmarks, such as angiogenesis and invasion, in gastric and other cancers 33-35. Higher HIF-1α expression in tumor specimens has also been associated with greater aggressiveness and poorer outcomes in patients receiving conventional treatment 36. Its central role in cancer metabolism has therefore made HIF-1α an attractive therapeutic target 37. Multiple approaches have been developed to interfere with HIF-1α regulation at different levels 38-42. These studies highlight the diverse mechanisms by which HIF-1α activity can be modulated, offering multiple avenues for therapeutic intervention in hypoxia-driven cancers. However, none have yet demonstrated broad clinical efficacy, largely due to issues with specificity, toxicity, and compensatory mechanisms in tumors. Belzutifan, a selective inhibitor of HIF-2α, represents the first successful clinical example of directly targeting hypoxia signaling in cancer therapy 43, 44. By preventing HIF-2α/HIF-1β heterodimerization, it suppresses transcription of hypoxia-responsive genes involved in angiogenesis and tumor growth. However, its clinical benefits have been largely restricted to tumors with specific genetic backgrounds, such as von Hippel-Lindau (VHL)-associated cancers, and it does not inhibit HIF-1α activity. Because HIF-1α is an important driver of glycolysis and malignant progression in solid tumors such as GC, alternative ways of modulating this pathway remain desirable. In this landscape, components of the OTUD4-FXR1-HIF-1α axis may represent alternative or complementary targets. FXR1 itself has been implicated as an oncogenic RNA-binding protein across multiple cancers and proposed as a potential target for anticancer therapy. Targeting FXR1 or upstream regulators such as OTUD4 could disrupt HIF-1α activation and its downstream metabolic effects, potentially overcoming some limitations of direct HIF-1α inhibition.

Overall, our findings expand the understanding of how deubiquitinase activity intersects with metabolic reprogramming in cancer progression and highlight the therapeutic potential of targeting OTUD4 and FXR1 in gastric cancer. Future work should explore pharmacological inhibition of OTUD4 and FXR1, potentially in combination with established metabolic or HIF1A pathway inhibitors, to assess synergy and clinical applicability. These efforts may yield new strategies to more effectively suppress tumor growth and metastasis in gastric cancer and other malignancies driven by aberrant ubiquitination and metabolic adaptation.

## Supplementary Material

Supplementary figures.

Supplementary tables.

## Figures and Tables

**Figure 1:**
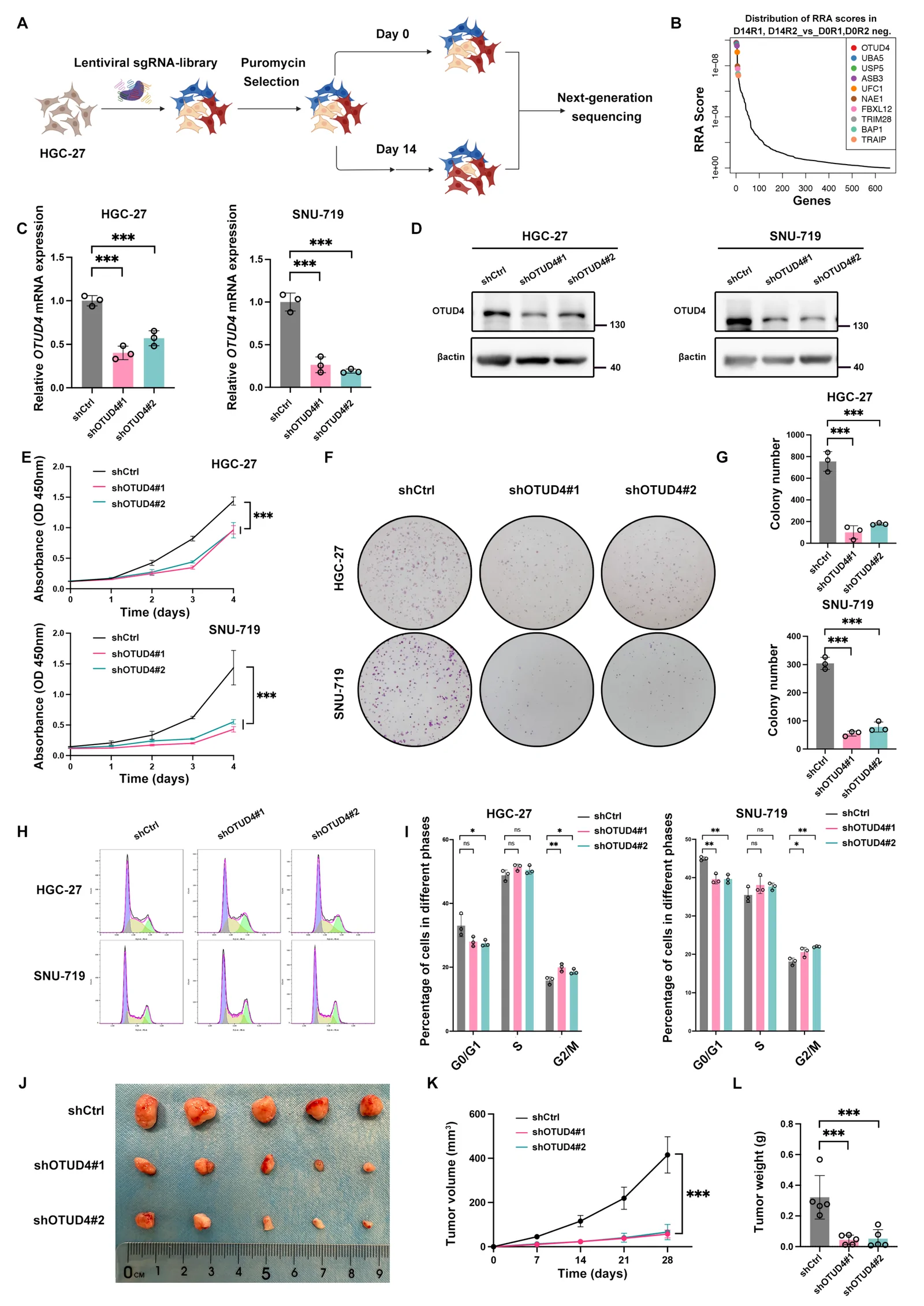
** OTUD4 promotes gastric cancer proliferation. A** Ubiquitin-Focused CRISPR screening identified genes potentially involved in regulating gastric cancer cell proliferation. **B** Top 10 candidate genes identified by RRA score analysis at day 14 relative to day 0.** C** Quantitative RT-PCR analysis confirmed OTUD4 knockdown in HGC-27 and SNU-719 cell lines with stable OTUD4 knockdown (n=3). **D** Western blot (WB) analysis confirmed OTUD4 knockdown in HGC-27 and SNU-719 cell lines with stable OTUD4 knockdown. **E** CCK-8 measurement of cell proliferation after OTUD4 knockdown in HGC-27 and SNU-719 cells (n=5). **F-G** Representative images of colony formation assays (**F**) and quantification of colony numbers (**G**) in OTUD4-knockdown and control gastric cancer (GC) cells (n=3). **H-I** Representative flow-cytometry plots (**H**) and quantitative cell-cycle analysis (**I**) on day 7 after shOTUD4 transfection (n=3). **J** Representative images of excised subcutaneous xenografts 4 weeks after inoculation (n=5). **K** Tumor growth curves of subcutaneous xenografts in the indicated groups (n=5). **L** Tumor weights measured at week 4 (n=5).

**Figure 2:**
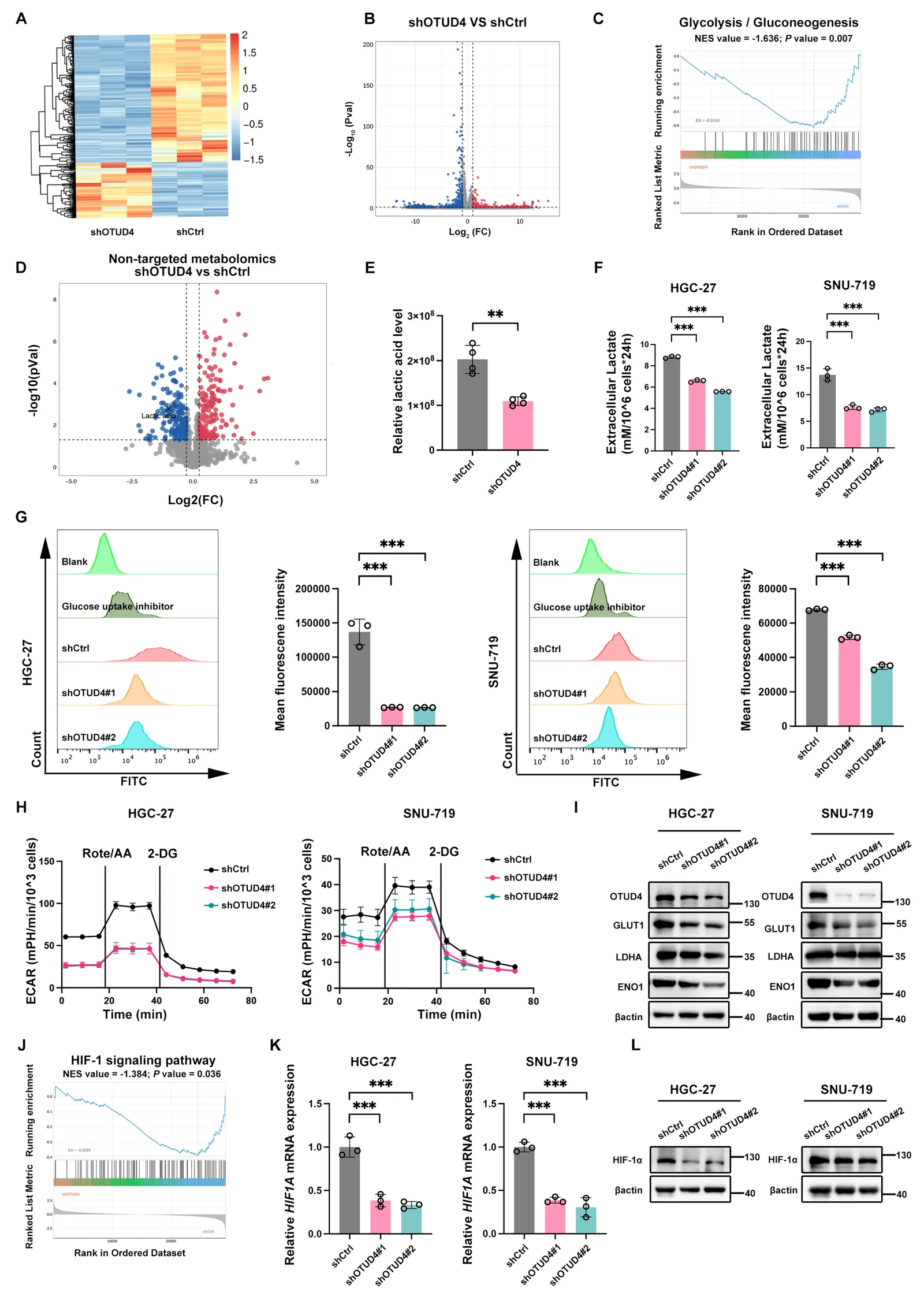
** OTUD4 enhances glycolysis in GC. A** Differentially expressed genes in SNU-719 cells carrying shOTUD4 or scramble control, displayed as a heatmap. **B** Volcano plot of gene expression differences between the indicated groups. **C** GSEA analysis revealed a reduction in glycolysis/gluconeogenesis following OTUD4 knockdown. **D** Volcano plot of differential metabolites in OTUD4-deficient and control SNU-719 cells (n=4). **E** Non-targeted metabolomics analysis revealed altered lactic acid levels in OTUD4-deficient compared with control SNU-719 cells (n=4). **F** Extracelluar lactate level of OTUD4-deficient and control GC cells (n=3). **G** Flow cytometric analysis of glucose uptake level in the indicated groups (n=3). **H** ECAR determined by Seahorse analysis in OTUD4-knockdown and control cells (n=3). **I** Immunoblotting of the glycolytic proteins GLUT1, LDHA, and ENO1 in the indicated groups. **J** GSEA demonstrating reduced HIF-1 signaling after OTUD4 knockdown. **K** *HIF1A* mRNA measured by qRT-PCR in control and OTUD4-deficient cells (n=3).** L** HIF-1α protein abundance in the indicated groups.

**Figure 3:**
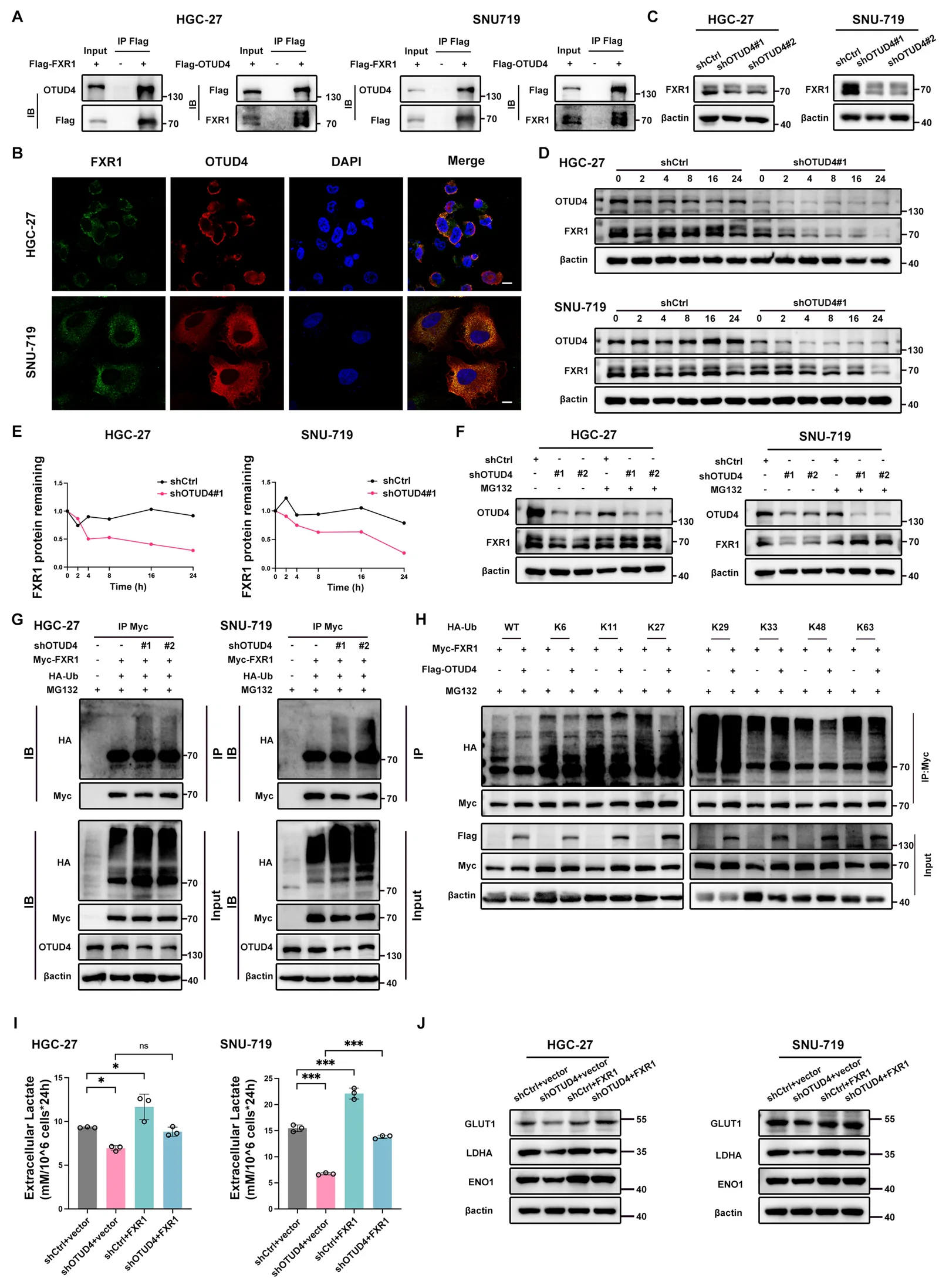
** FXR1 bridges OTUD4 and glycolytic regulation. A** Co-IP assessment of OTUD4-FXR1 association in the indicated groups. **B** IF detection of OTUD4 and FXR1 co-localization in HGC-27 and SNU-719 cells in HGC-27 and SNU-719 cells. Scale bar = 10µm. **C** FXR1 protein abundance in OTUD4-deficient and control GC cells. **D** FXR1 protein half-life was assessed in OTUD4-deficient and control GC cells after protein synthesis inhibitor CHX (50 μg/mL) treatment for the indicated times. **E** Quantification of FXR1 protein in HGC-27 and SNU-719 cells transduced with shCtrl or shOTUD4 after CHX treatment. **F** WB analysis of FXR1 protein level in OTUD4-deficient and control GC cells after proteasome inhibitor MG132 (10μM) treatment for 12h. **G** Ubiquitination of FXR1 in OTUD4-deficient and control cells reconstituted with Myc-FXR1 and HA-ubiquitin. **H** Effects of OTUD4 on the indicated FXR1 ubiquitin linkages. **I** Extracellular lactate level of OTUD4-knockdown HGC-27 and SNU-719 cells following transfection with FXR1 constructs or empty vector (n=3). **J** WB analysis of glycolysis related protein GLUT1, LDHA and ENO1 in the indicated groups.

**Figure 4:**
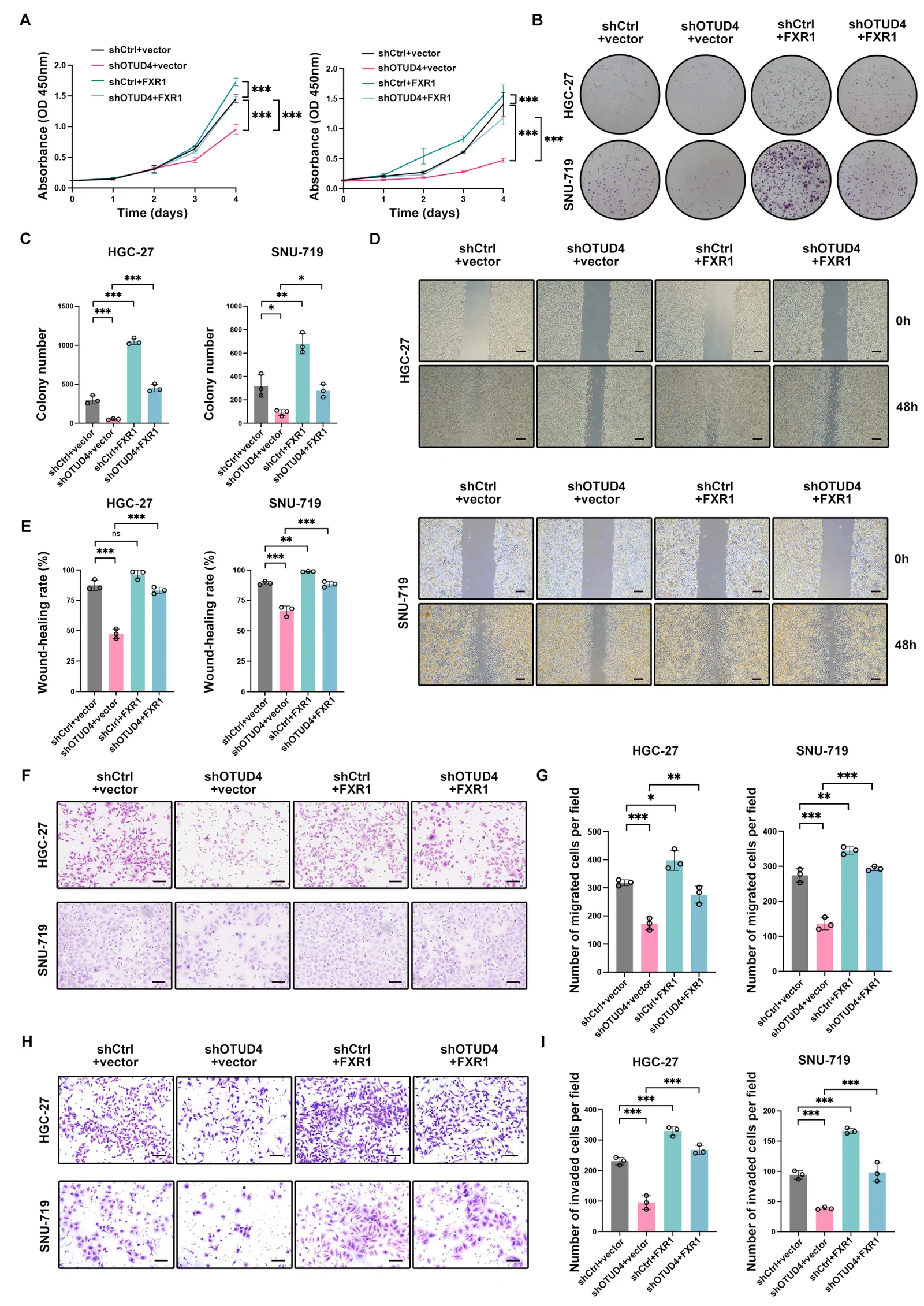
** OTUD4 facilitates gastric cancer progression via FXR1 *in vitro*. A** CCK-8 assay of proliferation in OTUD4-knockdown HGC-27 and SNU-719 cells after introduction of FXR1 constructs or empty vector (n=5). **B-C** Representative pictures (**B**) of colony formation assay and statistics (**C**) of colony number in the indicated groups (n=3). **D-E** Representative images (**D**) and quantification (**E**) of the wound-healing migration assays in the indicated groups. Scale bar = 1mm (n=3). **F-G** Representative images (**F**) and quantification (**G**) of migrated cells in Transwell migration assays in the indicated groups. Scale bar = 100µm (n=3). **H-I** Representative images (H) and quantification (I) of invaded cells in Transwell invasion assays in the indicated groups. Scale bar = 100µm (n=3).

**Figure 5:**
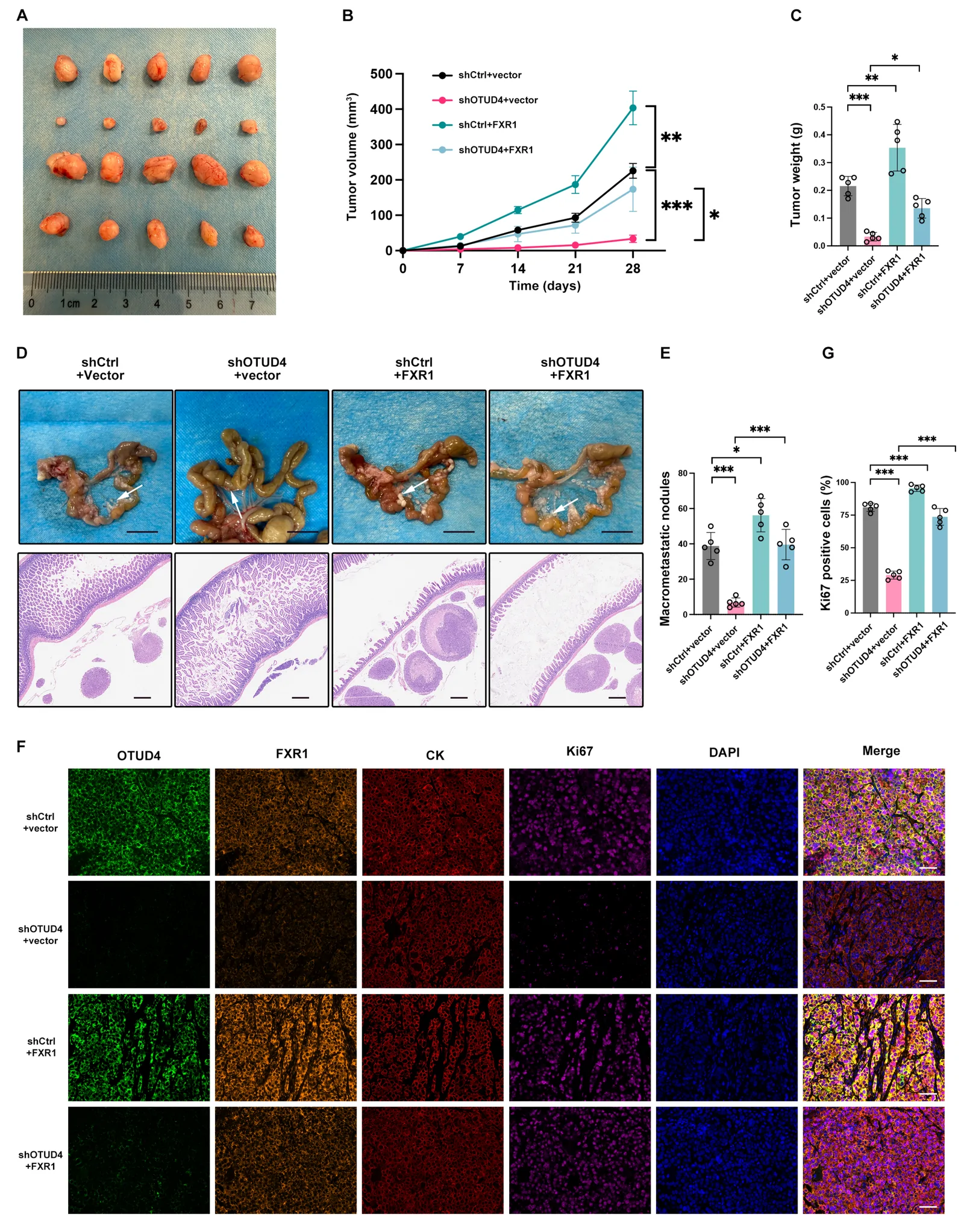
** OTUD4 facilitates gastric cancer progression via FXR1 *in vivo*. A** Image of dissected subcutaneous xenografts in OTUD4-knockdown MKN45 cells following transfection with FXR1 constructs or empty vector (n=5). **B** Tumor growth trajectories for the indicated groups (n=5). **C** Xenograft weights at 4 weeks after inoculation (n=5). **D** Mesenteric metastatic nodules (upper) and corresponding HE-stained sections (lower) from the indicated groups (n=5). Arrows indicate metastatic nodules. Upper scale bar = 10mm, Lower scale bar = 500µm. **E** Quantification of macro metastatic nodules (n=5). **F** Representative photos of mIHC staining for OTUD4, FXR1, CK (cancer cell marker), and Ki67 (proliferation marker) in the indicated groups. Scale bar = 50µm. **G** Ki67-positive cell counts determined by mIHC (n=5).

**Figure 6:**
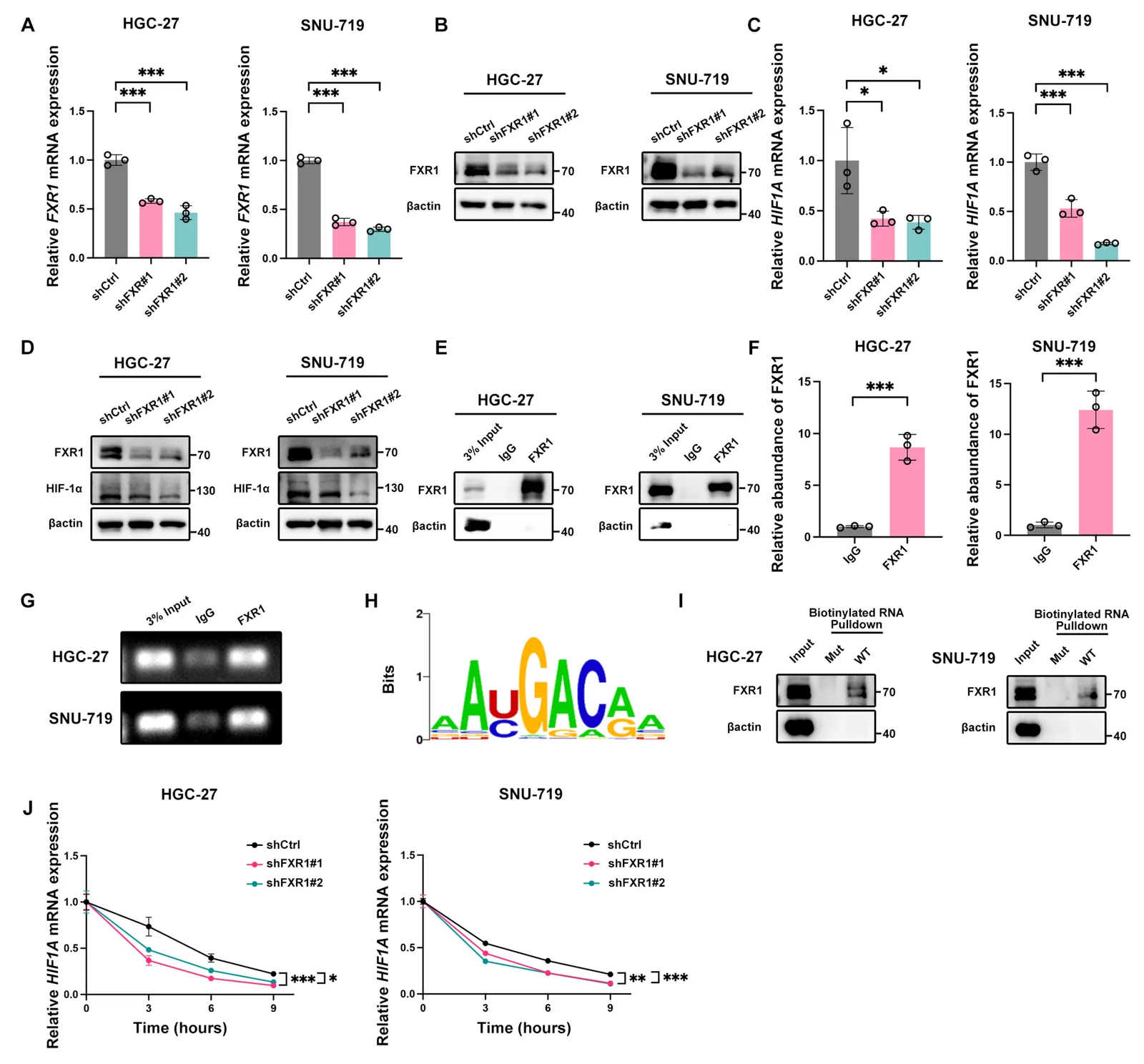
** FXR1 promotes glycolysis through stabilization of *HIF1A* mRNA. A-B** Confirmation of FXR1 depletion at the mRNA (**A**) and protein (**B**) levels in HGC-27 and SNU-719 cells with stable FXR1 knockdown (n=3). **C-D** HIF-1α expression measured at the transcript (**C**) and protein (**D**) levels in FXR1-deficient and control GC cells (n=3). **E** Immunoblot detection of FXR1 in RIP samples from HGC-27 and SNU-719 cells. **F-G** qRT-PCR (**F**) and agarose gel electrophoresis (**G**) showing FXR1 association with *HIF1A* mRNA by RIP (n=3). **H** The recognition motif of FXR1 predicted by RBPsuite. **I** WB analysis of FXR1 pulled down by biotinylated *HIF1A* mRNA. **J** *HIF1A* mRNA levels in FXR1-deficient and control cells after actinomycin D (5 μg/mL) treatment for the indicated times (n=3).

**Figure 7:**
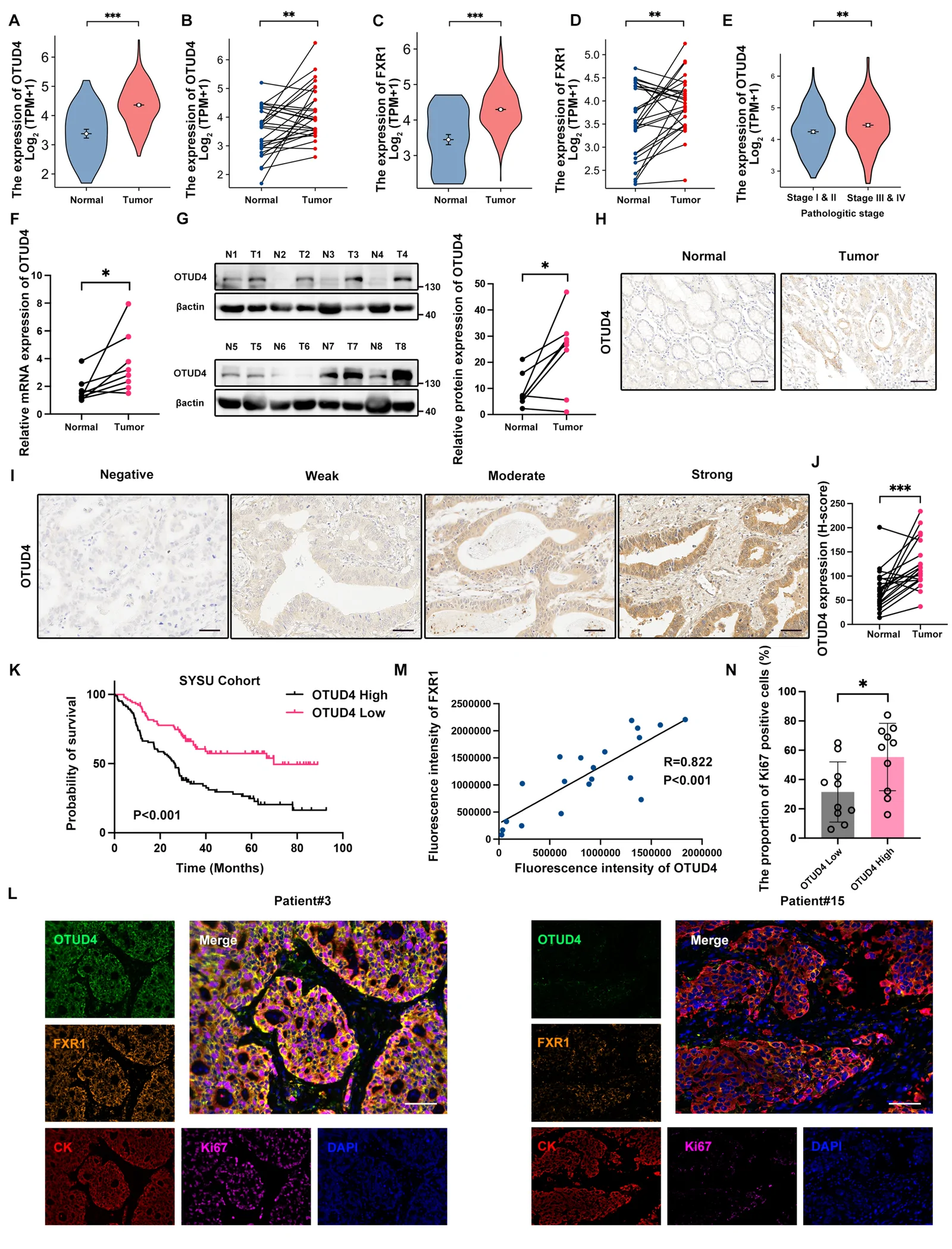
** OTUD4 is overexpressed in gastric cancer and serves as a predictor of poor patient outcomes. A-B** OTUD4 expression in unmatched (**A**) and matched (**B**) GC TCGA samples. **C-D** FXR1 expression in unmatched (**C**) and matched (**D**) GC TCGA samples. **E** OTUD4 expression in advanced versus earlier-stage GC based on TCGA data. **F-G** OTUD4 mRNA (**F**) and protein (**G**) levels in GC versus normal gastric tissues (n=8). **H** Representative IHC images of OTUD4 expression in gastric cancer and corresponding normal gastric tissues (n = 20). Scale bar=50 µm. **I** Representative IHC images illustrating negative, weak, moderate, and strong OTUD4 staining in GC patients. Scale bar = 50 µm. **J** H-score comparison of OTUD4 in gastric cancer and normal tissues (n=20). **K** Kaplan-Meier overall-survival curves. **L** Representative mIHC staining for OTUD4, FXR1, CK, and Ki67 (n=20). Scale bar = 50µm. **M** Correlation between OTUD4 and FXR1 fluorescence intensities measured by mIHC. **N** Ki67-positive fractions according to OTUD4 status.

## Data Availability

The RNA-seq data generated in this study was deposited in the Sequence Read Archive (SRA) under accession number PRJNA1419148. All other data supporting the findings of this study are available from the corresponding author upon reasonable request.
