# Supplementary material for: Glycolytic Reprogramming Mediated by the OTUD4-FXR1-HIF1A Axis Drives Gastric Cancer Progression
Source: Int J Biol Sci. 2026 Sep 3;22(14):7845–61. doi: 10.7150/ijbs.138384 (PMC13617998; doi:10.7150/ijbs.138384)
Supplement: Supplementary file 1 [file ijbsv22p7845s1.pdf]

Glycolytic Reprogramming Mediated by the OTUD4–FXR1–HIF1A Axis Drives Gastric Cancer Progression

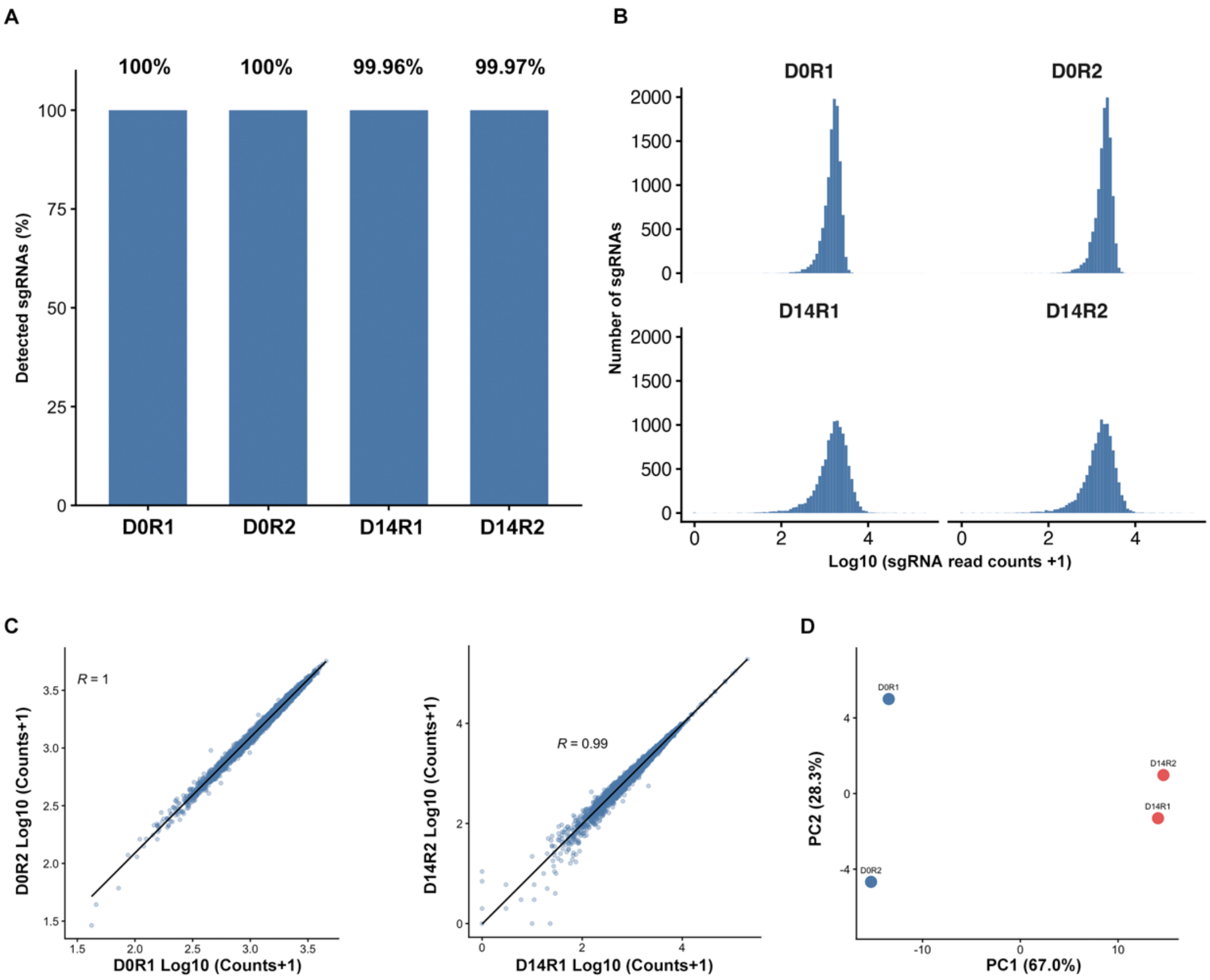

Fig. S1 Quality control of the CRISPR screening library. A Percentage of detected sgRNAs across samples collected at day 0 and day 14, including two biological replicates at each time point (D0R1, D0R2, D14R1, and D14R2). **B** sgRNA read count distribution across the indicated samples. **C** Correlation analysis between biological replicates. **D** Principal component analysis of the indicated samples.

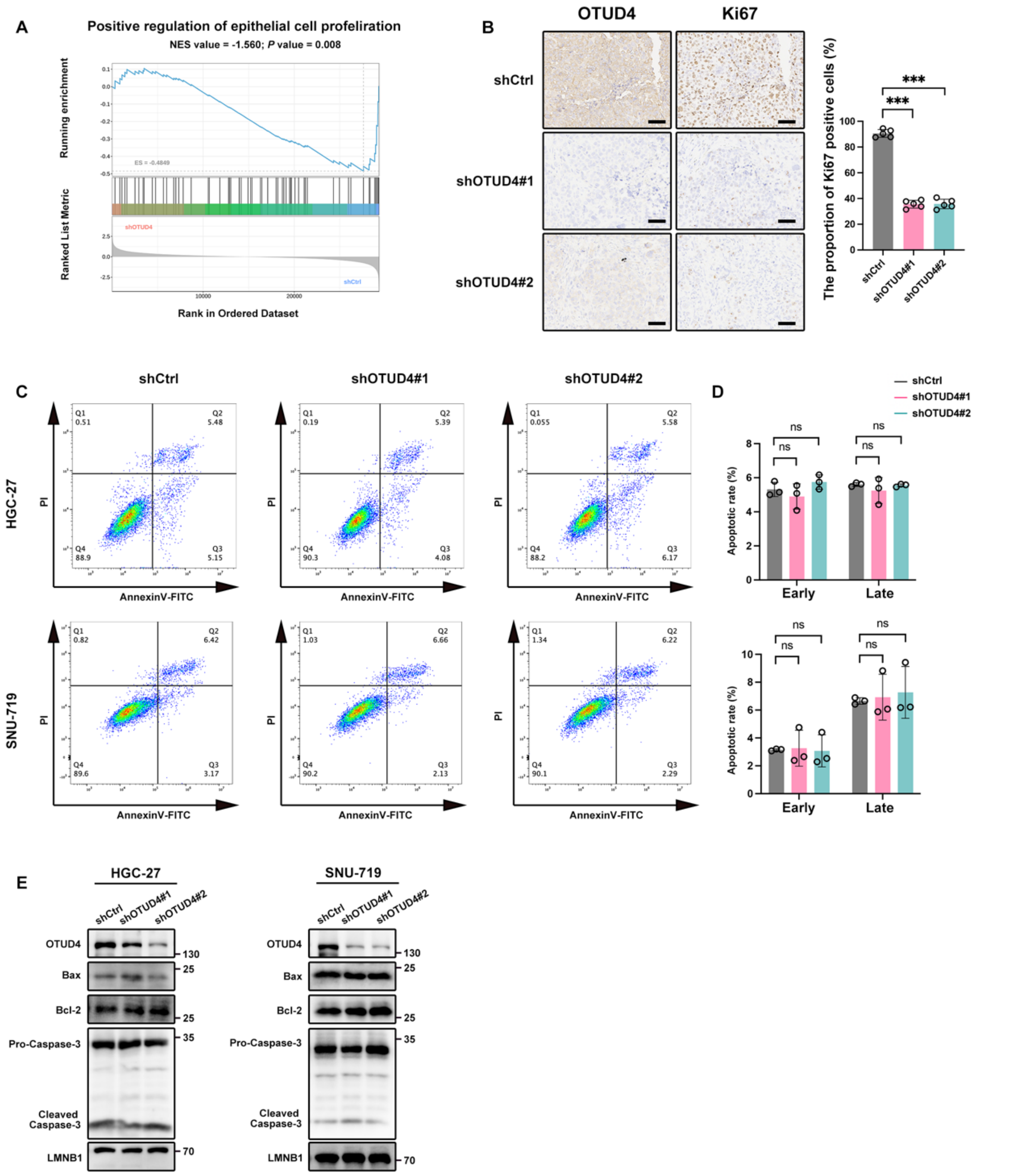

Fig.S2 OTUD4 enhances gastric cancer cell proliferation without influencing apoptosis. A GSEA analysis shows a reduction in epithelial cell proliferation following OTUD4 knockdown. **B** Representative IHC images and quantitative analysis of Ki67 expression in OTUD4 knockdown and control subcutaneous xenografts tumor (n=5). Scale bar = 50μm. **C** Flow cytometric analysis of apoptosis in the OTUD4 knockdown

compared with control cells (n=3). **D** Quantification of apoptotic rates in the indicated groups by flow cytometry (n=3). **E** WB analysis of apoptotic-related protein Bax, BCL-2 and Caspase-3 in the indicated groups.

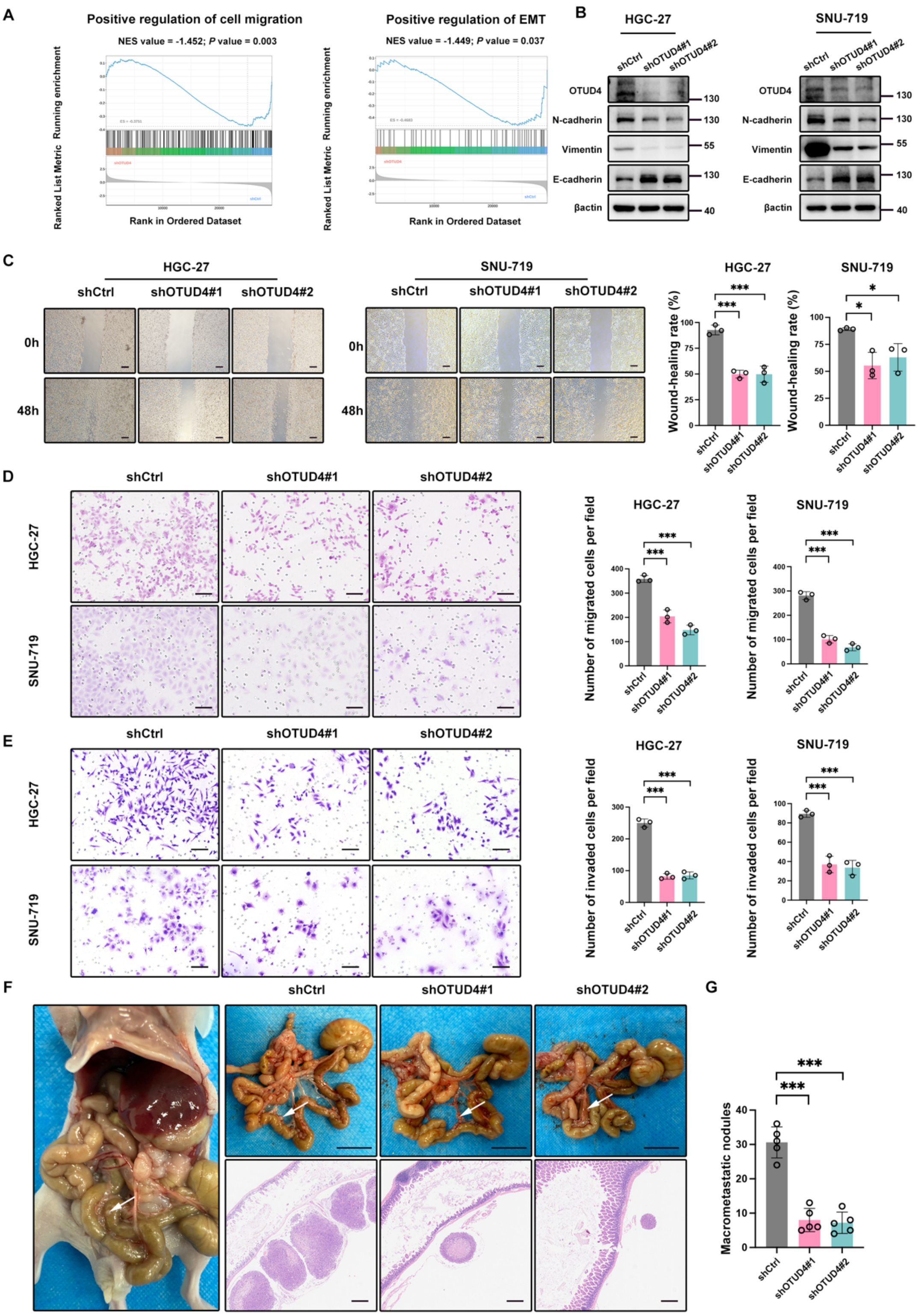

Fig.S3 OTUD4 promotes gastric cancer migration and invasion. A GSEA analysis indicated decreased cell migration (left) and EMT level (right) in SNU-719 cells following OTUD4 knockdown. **B** WB analysis revealed altered expression of EMT-

related proteins, including N-cadherin, Vimentin, and E-cadherin, in OTUD4-knockdown HGC-27 and SNU-719 cells compared with control cells. **C** Representative images and quantification of OTUD4 knockdown and control GC cells in the wound-healing migration assays (n=3). Scale bar = 1mm. **D** Representative images and quantification of the indicated groups in the Transwell migration assays (n=3). Scale bar = 100µm. **E** Representative images and quantification of the indicated groups in the Transwell invasion assays (n=3). Scale bar = 100µm. **F** Representative images (upper) and hematoxylin and eosin (HE) staining (lower) of mesenteric metastatic nodules (arrows). Upper scale bar = 10mm, Lower scale bar = 500µm. **G** Quantitative analysis of macro metastatic nodules in the indicated groups (n=5).

Fig.S4 OTUD4 enhances glycolysis in GC. KEGG analysis of top 30 *P* value of GSEA enrichment.

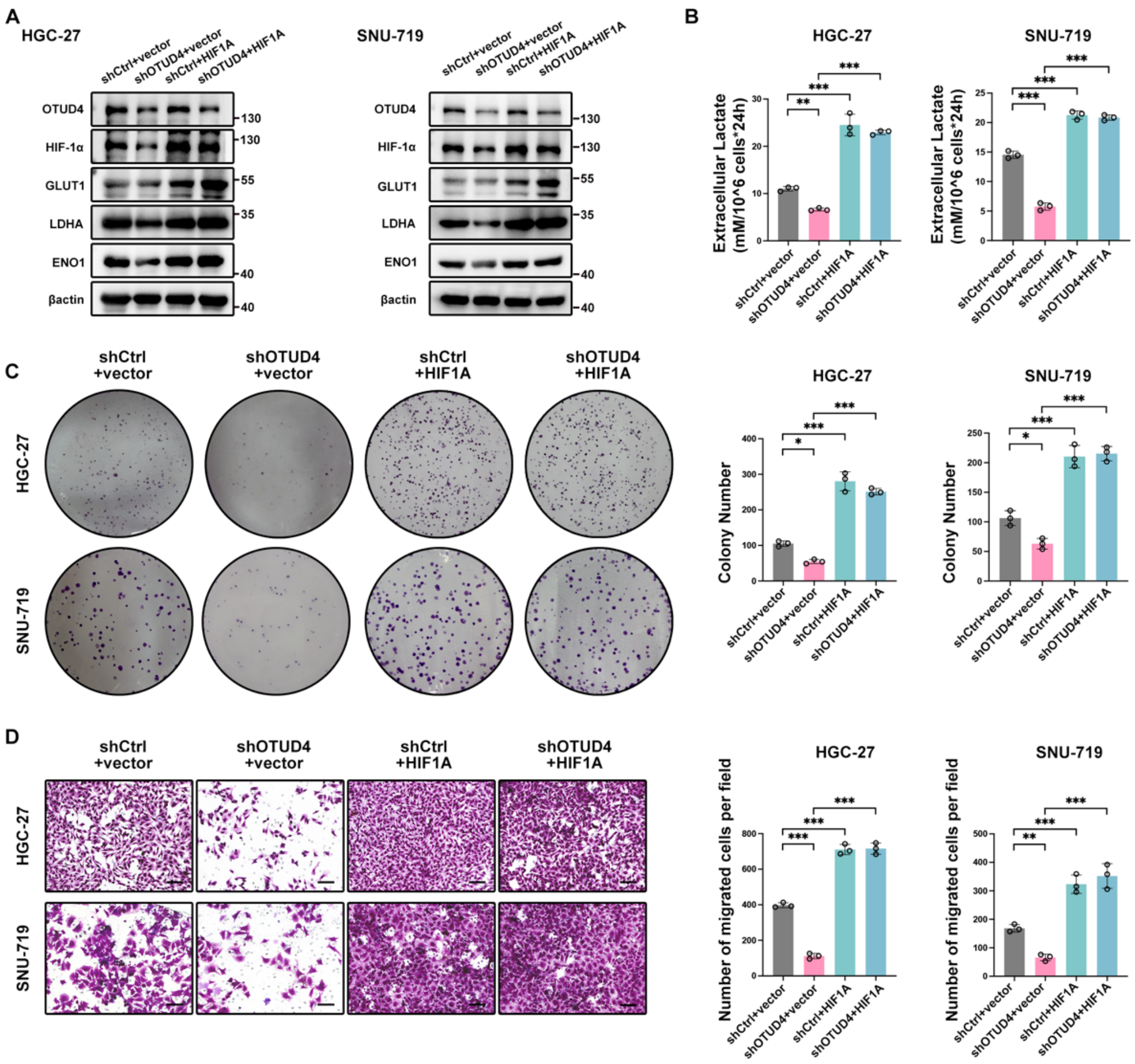

Fig.S5 HIF1A overexpression rescues glycolysis and malignant phenotypes suppressed by OTUD4 knockdown. A WB analysis of glycolysis related protein GLUT1, LDHA and ENO1 in OTUD4-knockdown HGC-27 and SNU-719 cells following transfection with HIF1A constructs or empty vector. **B** Extracellular lactate level in the indicated groups. **C** Representative images of colony formation assays and quantification of colony numbers in the indicated groups. **D** Representative images and quantification of Transwell migration assays in the indicated groups (n=3). Scale bar = 100μm.

Fig.S6 OTUD4 stabilizes FXR1 by mediating its deubiquitination. A Co-IP assays showed that OTUD4 did not interact with HIF-1α in HGC-27 and SNU-719 cells. **B** Immunoblotting revealed that the N-terminal region (amino acids 1–245) of OTUD4 mediates its interaction with FXR1.

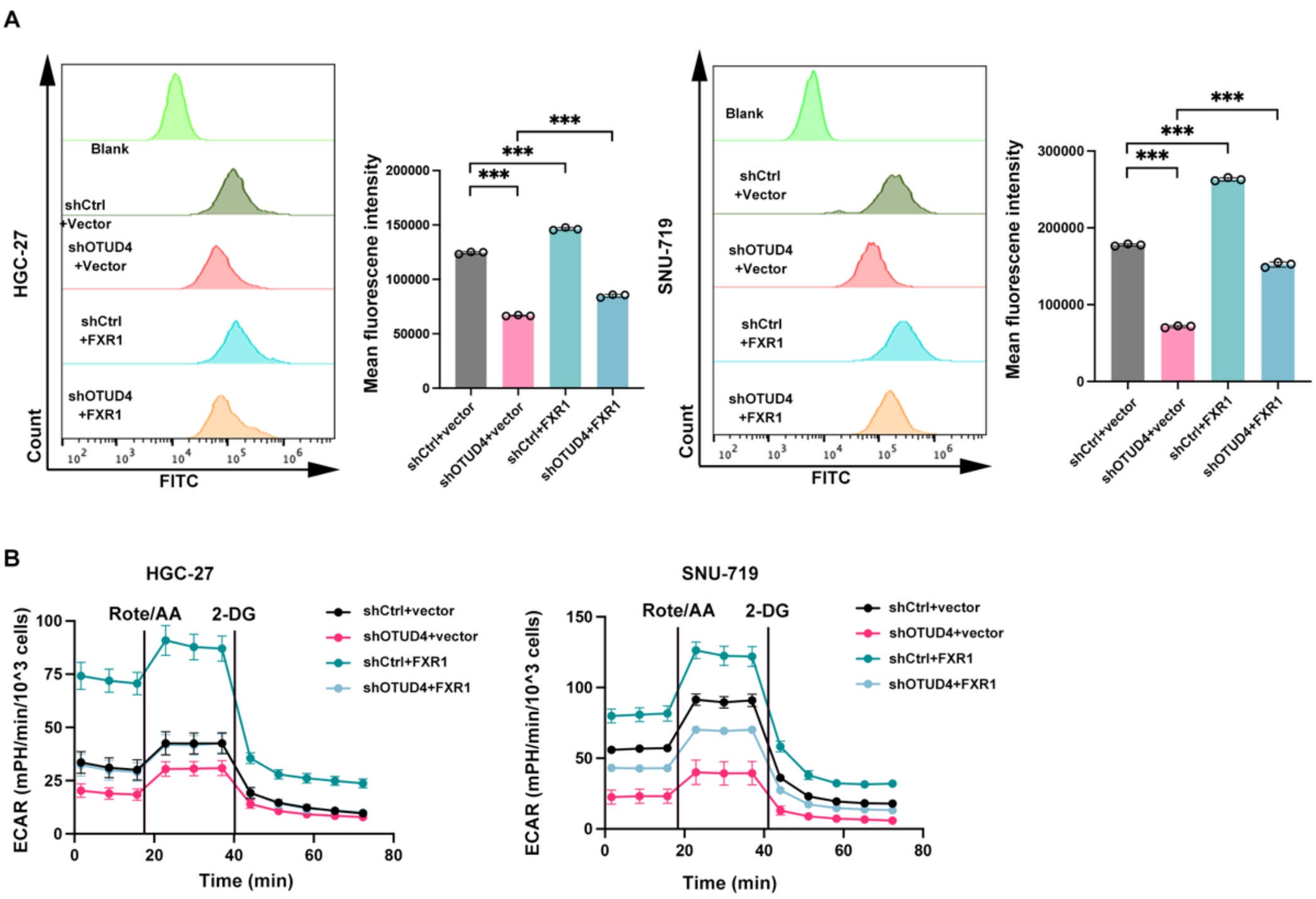

Fig.S7 OTUD4 enhances glycolysis in a FXR1-dependent manner. A Flow cytometric analysis of glucose uptake level in OTUD4-knockdown HGC-27 and SNU-719 cells following transfection with FXR1 constructs or empty vector, quantified as mean fluorescence intensity (MFI, n=3). **B** Seahorse analysis of ECAR in the indicated groups.

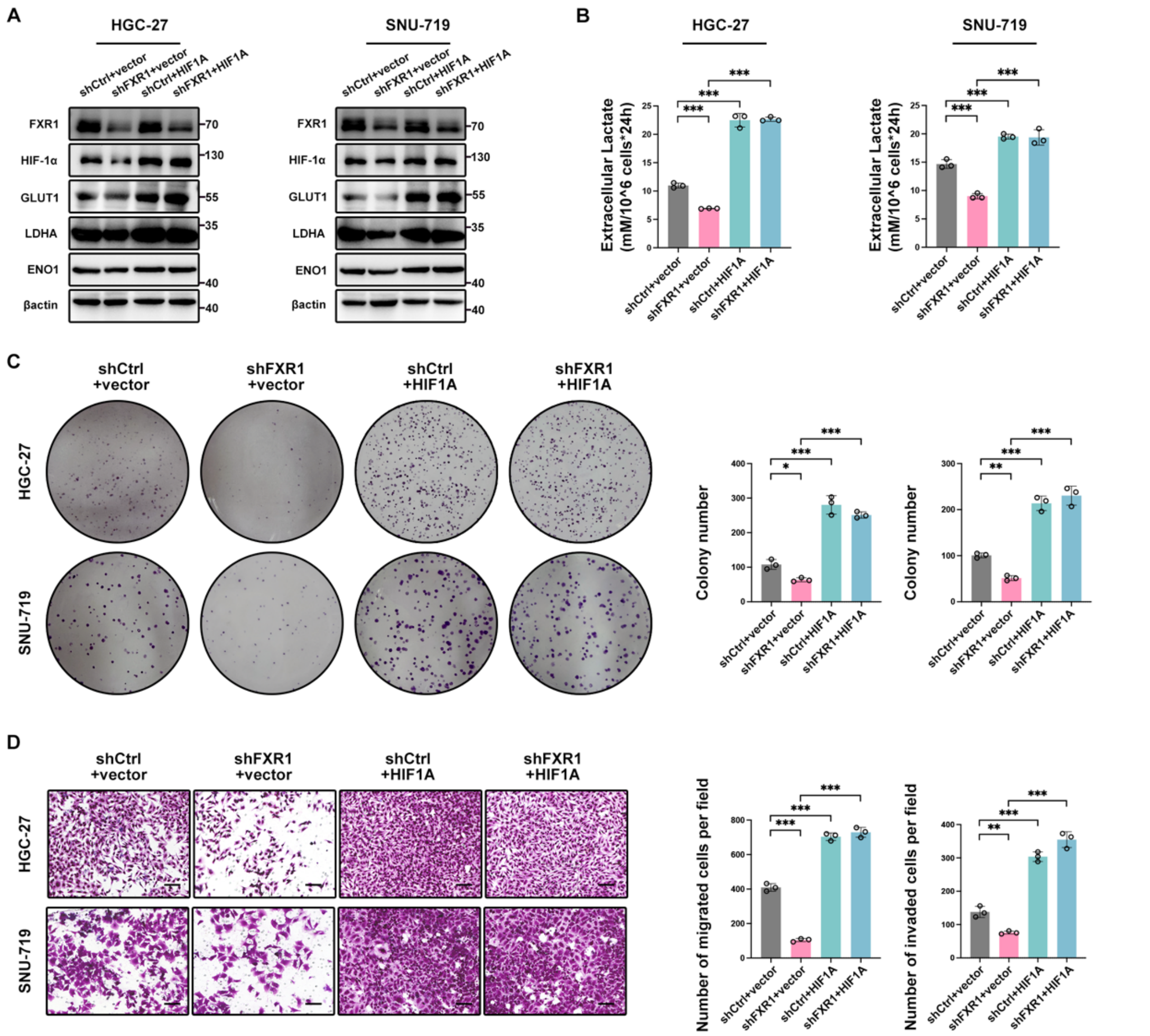

Fig.S8 HIF1A overexpression rescues glycolysis and malignant phenotypes suppressed by FXR1 knockdown. A WB analysis of glycolysis related protein GLUT1, LDHA and ENO1 in FXR1-knockdown HGC-27 and SNU-719 cells following transfection with HIF1A constructs or empty vector. **B** Extracellular lactate level in the indicated groups. **C** Representative images of colony formation assays and quantification of colony numbers in the indicated groups. **D** Representative images and quantification of Transwell migration assays in the indicated groups (n=3). Scale bar = 100μm.

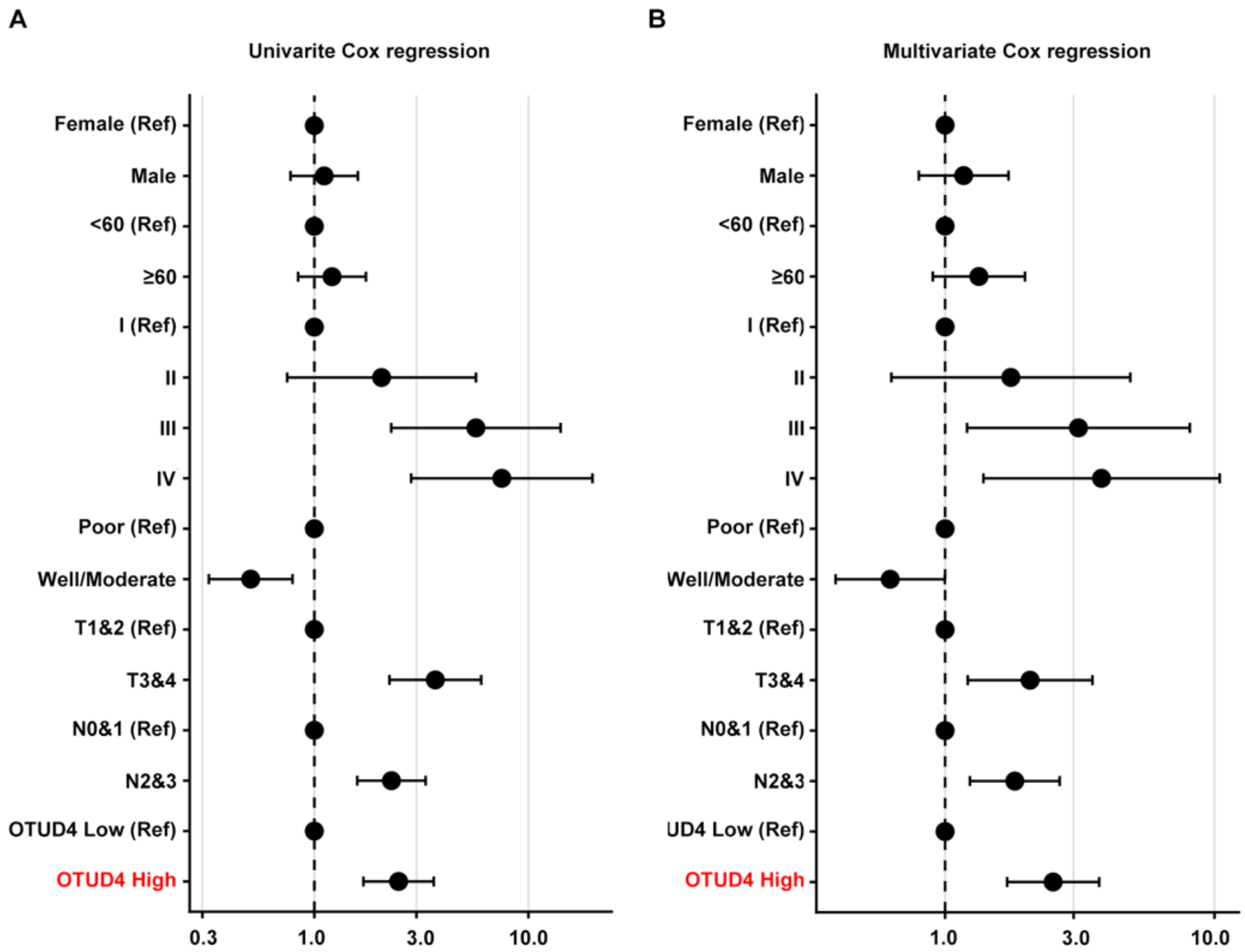

Fig.S9. Univariate and multivariate Cox regression analyses of overall survival in patients with gastric cancer. **A** Univariate Cox regression analysis of OTUD4 expression and clinicopathological variables. **B** Multivariate Cox regression analysis of OTUD4 expression and clinicopathological variables. Forest plots show hazard ratios (HRs), 95% confidence intervals (CIs).
